# Comprehensive analysis of three female patients with different types of X/Y translocations and literature review

**DOI:** 10.1186/s13039-023-00639-z

**Published:** 2023-05-18

**Authors:** Shanquan Liu, Jiemei Zheng, Xijing Liu, Yi Lai, Xuan Zhang, Tiantian He, Yan Yang, He Wang, Xuemei Zhang

**Affiliations:** 1 Department of Medical Genetics & Prenatal Diagnosis Center, West China Second University Hospital, Sichuan University, No.20, South Section 3, Renmin Road, Chengdu, 610041 Sichuan China; 2 Department of Obstetrics & Gynecology, West China Second University Hospital, Sichuan University, Chengdu, China; 3 Key Laboratory of Birth Defects and Related Diseases of Women and Children(Sichuan University), Ministry of Education, Chengdu, China

**Keywords:** X/Y translocation, Clinical and genetic analysis, Literature review, Re-classified

## Abstract

**Background:**

X/Y translocations are highly heterogeneity in terms of clinical genetic effects, and most patients lack complete pedigree analysis for clinical and genetic characterization.

**Results:**

This study comprehensively analyzed the clinical and genetic characteristics of three new patients with X/Y translocations. Furthermore, cases with X/Y translocations reported in the literature and studies exploring the clinical genetic effects in patients with X/Y translocations were reviewed. All three female patients were carriers of X/Y translocations with different phenotypes. The karyotype for patient 1 was 46,X,der(X)t(X;Y)(p22.33;q12)mat, patient 2 was 46,X,der(X)t(X;Y)(q21.2;q11.2)dn, and patient 3 was 46,X,der(X)t(X;Y)(q28;q11.223)t(Y;Y)(q12;q11.223)mat. C-banding analysis of all three patients revealed a large heterochromatin region in the terminal region of the X chromosome. All patients underwent chromosomal microarray analysis, which revealed the precise copy number loss or gain. Data on 128 patients with X/Y translocations were retrieved from 81 studies; the phenotype of these patients was related to the breakpoint of the chromosome, size of the deleted region, and their sex. We reclassified the X/Y translocations into new types based on the breakpoints of the X and Y chromosomes.

**Conclusion:**

X/Y translocations have substantial phenotypic diversity, and the genetic classification standards are not unified. With the development of molecular cytogenetics, it is necessary to combine multiple genetic methods to obtain an accurate and reasonable classification. Thus, clarifying their genetic causes and effects promptly will help in genetic counseling, prenatal diagnosis, preimplantation genetic testing, and improvement in clinical treatment strategies.

## Background

X/Y translocation is a rare chromosomal abnormality, with approximately 128 patients reported in the literature [1–11], most of which are limited to single patient reports and a few comprehensive analyses of complete families. Aberrations in sex chromosomes can affect gonadal development, leading to complex reproductive and endocrine disorders. In females, it may affect ovarian function and uterine development. In males, it can lead to azoospermia, cryptorchidism, penis or testicular hypoplasia, or it may lead to ovotesticular disorders of sex development [12], gynecomastia [10, 11, 13–15]. Although it has been reported in both men and women at birth [2], later diagnoses also occur, which limits possible medical treatments and surgical intervention [16]. Additionally, several studies proved that the presence of Y chromosome material in females leads to an increased risk of gonadoblastoma (10 to 30%) [17, 18].

There are many types of X/Y translocations. Hsu (1994) divided the category of Y/X translocations with der(X) into seven types and further classified the category of Y/X translocations with der(Y) into four types [1]. However, these classifications are complex and cannot be applied to current clinical cytogenetics.

This study conducted a comprehensive analysis of the clinical phenotypes, cytogenetics, and molecular genetics of three patients with rare X/Y translocations and their family members. We also reviewed the relevant published literature, exploring the origin of derivative chromosomes produced by X and Y translocations and the phenotype and fertility of patients carrying this derived chromosome. In addition, we provide suggestions on genetic counseling for patients and reclassify the X/Y translocations into new types based on the breakpoints of the translocated X and Y chromosomes.

## Materials and methods

### Patients

Three patients and their family members were recruited from the West China Second University Hospital. The patients visited our hospital for infertility or menstrual disorders. The present study was approved by the Medical Ethics Committee of West China Second University Hospital, Sichuan University. Informed consent to participate in the study was obtained from all participants.

### Methods

#### Chromosome G-banding karyotype analysis

Peripheral blood (5 ml) was drawn into a heparin anticoagulant tube. Blood samples were processed according to *The Association of Genetic Technologists (AGT) cytogenetic laboratory procedures* and analyzed for peripheral blood lymphocyte karyotyping [19]. The diagnosis and results complied with the relevant standards of the *International System for Human Cytogenomic Nomenclature, 2020 (ISCN, 2020)*. The chromosome image analysis system used for karyotyping and image capturing was a Metafer-Automated Slide Scanning Platform and Ikaros from Zeiss.

#### Chromosome C-banding analysis

Chromosome C-banding preparation was performed according to *The AGT cytogenetic laboratory procedures* [19]. The chromosome dispersion was good and the length was suitable for splitting the middle chromosome image under a 100 × 100 microscope and image capture.

#### Fluorescence in situ hybridization (FISH)

FISH was used to analyze the sex chromosome centromeres. AneuVysion probe sets (Vysis/Abbott, Downers Grove, IL, USA) were used for hybridization of metaphase cells according to the manufacturer’s protocol, and the analysis was based on the latest American College of Medical Genetics and Genomics (ACMG) guidelines [20].

#### Chromosomal microarray analysis (CMA)

Single nucleotide polymorphism-chromosomal microarray analysis was performed using Affymetrix CytoScan 750 K Array chips and Affymetrix Chromosome Analysis Suite (ChAS) software, and the detected structure was analyzed according to the latest ACMG guidelines [21].

#### Literature review

A literature review was conducted through a targeted search for case reports and original articles on X/Y translocation in English journals archived in PubMed (https://www.ncbi.nlm.nih.gov/pubmed/) and Chinese medical journals archived in the China National Knowledge Infrastructure CNKI (http://www.cnki.net) and WANFANG DATA (http://www.wanfangdata.com), from January 1978 to April 2022.

## Results

### Case presentation

**Fig. 1:**
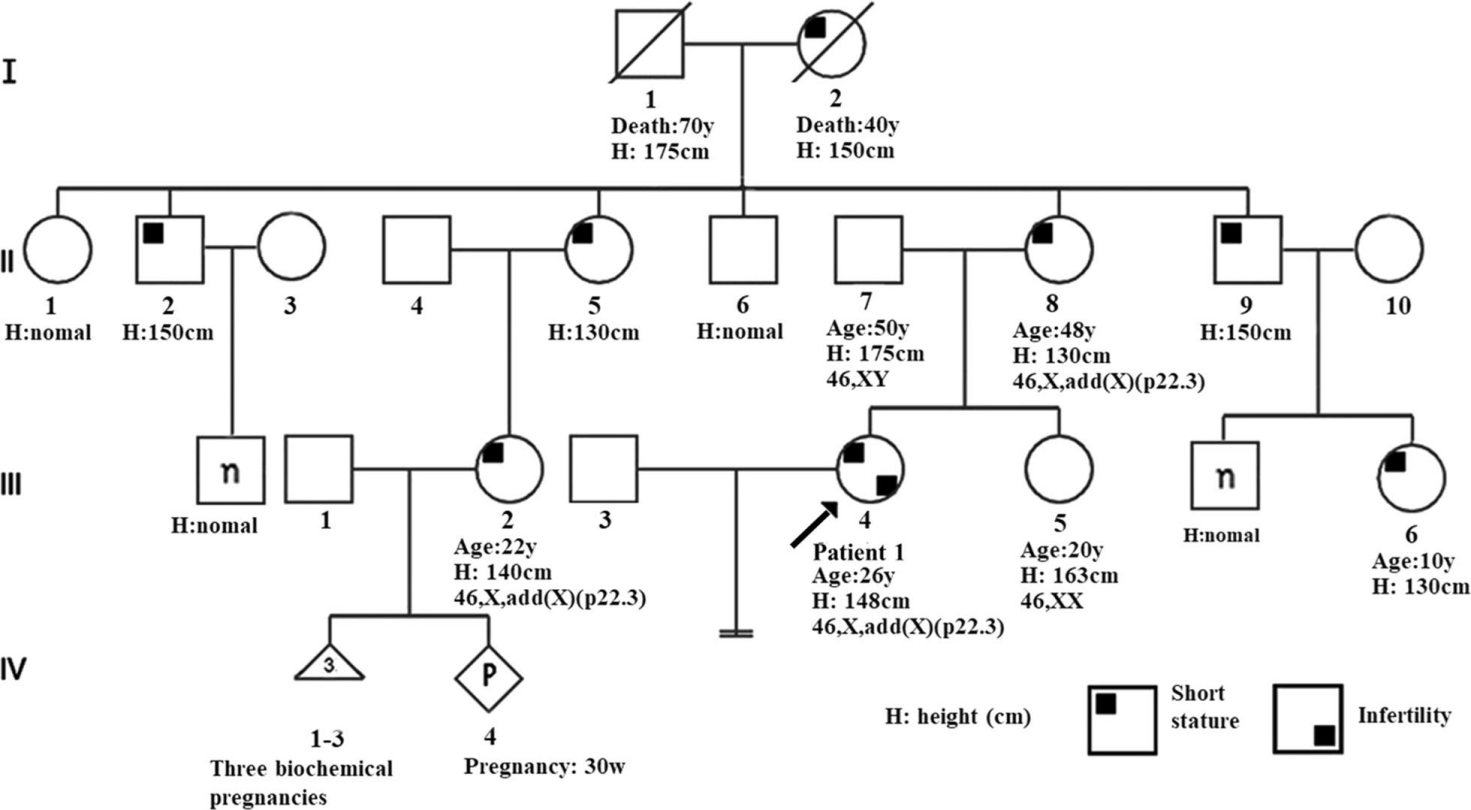
Pedigree of patient 1. The height of the relevant family members is marked in the figure, showing that most of family members had short stature. Five individuals had chromosomal karyotype results, and three members with short stature(II-8, III-2, and III-4) had the same 46,X,add(X)(p22.3) as the proband, while an individual with normal height(II-8) has normal male karyotype

*Patient 1* (Fig. 1, III-4) was a female (age: 26 years, height: 148 cm, and weight: 44 kg) who was admitted to our hospital for infertility after five years of normal sexual life. When she was 13 years old, her height was about 120 cm, which was shorter than that of her peers (− 2 SD), but no treatment was provided; the age at menarche was 13 years, and menstrual cycle was normal. At the age of 25, she underwent preimplantation genetic testing (PGT) at another hospital; 10 eggs were harvested, only one embryo was obtained, and implantation failed. There was no consanguinity in her family, several members were short in stature (140–150 cm; Fig. 1), and none had intellectual disabilities. Two of her cousins (Fig. 1, III-2, III-6) were short in stature, and one (Fig. 1, III-2) had had three biochemical pregnancies and was at 30 weeks of pregnancy at the time of reporting. Detailed clinical information is presented in Table 1. Ultrasonography of the uterine adnexa showed no significant abnormalities. Sex hormone testing showed estradiol (E2): 45.03; progesterone (P): 0.26; luteinizing hormone (LH): 4.34; follicle-stimulating hormone (FSH): 7.34; and Anti-Müllerian Hormone (AMH) (ELISA): 1.96 ng/ml. Clinical materials and laboratory test results are summarized in Table 1. Currently, the patient is planning to undergo adjuvant fertility therapy.

**Table 1:** Summary of clinical data and genetic test results for three cases

Cases		Patient 1	Patient 2	Patient3
Age (year)		26	21	23
Height (cm)		148	168	150
Weight (kg)		44	57	54
Mainly complaints		Primary infertility	Menstrual disorders, secondary amenorrhea	Primary infertility
Sex horm-ones	FSH(IU/L) (2.5–10.2)	7.34	68.9↑	3.4
	LH(IU/L) (1.9–12.5)	4.34	24.2	5.1
	T (ng/ml) (0.1–0.5)	0.03↓	0.23	0.18
	E2(pg/ml) (19.5–144.2)	45.03	< 11.8↓	102.3
	P (ng/ml) (0.15–1.40)	0.26	0.59	15.29
	PRL(ng/ml) (2.8–29.2)	10.48	8.7	14.9
AMH(ELISA)(ng/ml) (2.5–6.3)		1.96↓	< 0.06↓	3.97
Ultrasound		Normal uterus (4.3 × 4.5 × 4.4 cm); both ovaries are normal, and left ovaries has 2–3 follicles inside, maximum diameter 0.7 cm; normal bilateral fallopian tubes	Small uterus (the diameter of uterus is 2.4 cm), the endometrial thickness is about 0.15 cm (single layer); bilateral streak ovaries	Normal uterus (3.6 × 4.0 × 4.1 cm); both ovaries are normal; non-visualized bilateral fallopian tubes (surgical removaled)
History of pregnancy and childbirth		G0P0	G0P0	G0P0
Family		Some family members have short stature, the mother and cousin’s G-banding karyotypes are same as that of the patient, while the father and sister’s karyotype are normal	Parents are consanguineous, with normal phenotypes, and normal G-banding karyotype	The mother had the same karyotype as the patient; and had three miscarriages. The father, sister and two uncles had normal G-banding karyotype,
Genetic test results		46,X,der(X)t(X;Y)(p22.33;q12)mat.arr[GRCh38]Xp22.33 (251888_1772154) × 1(1520 kb)	46,X,der(X)t(X;Y)(q21.2;q11.2)dn.arr[GRCh38]Xq21.2q28(86025630_155714301) × 1,Yq11.222q12(18443276_26653507) × 1	46,X,der(X)t(X;Y)(q28; q11.223)t(Y;Y)(q12;q11.223)mat. arr[GRCh38]Xq28(155077922_155700385) × 1,Yq11.223q12(21924024_26653507) × 2

*FSH* Follicle stimulating hormone, *LH* Luteinizing hormone, *T* Testosterone, *E2* Estradiol, *P* Progesterone, *PRL* Prolactin, *AMH* Anti-Müllerian hormone

**Fig. 2:**
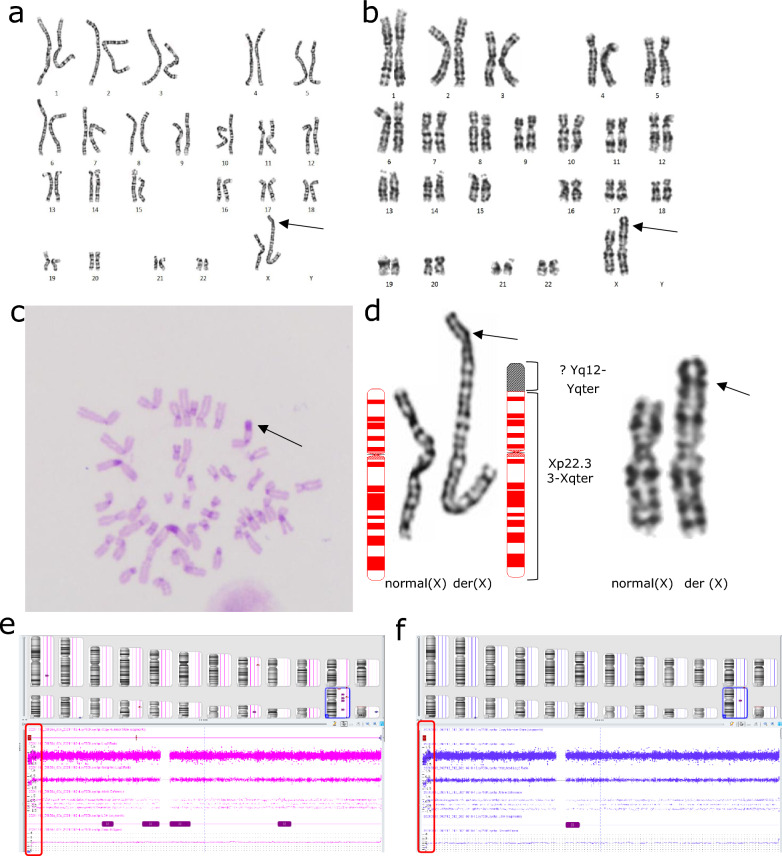
Cytogenetic and CMA results of patient 1, her mother and her cousin. **a**: G-banded karyogram (550 bands) of the patient, showing der(X) chromosome; **b**: G-banded karyogram (400 bands) of the patient’s mother, showing the same der(X) as patient 1; **c**: C-banded metaphase spread showing a large heterochromatin segment (indicated by an arrow) at the end of short arm of the der(X), which is suspected to be a part of chromosome Yq; **d**: Comparison and pattern representation of patient 1 (left) and her mother’s (right) X chromosomes. **e**: The results of CMA for patient 1 indicate the deletion of large segment of the X chromosome of 1.52 Mb (red box in e); **f**: The results of the CMA for patient 1’s cousin (III-2) is the same as that of patient 1 (red box), and no Y chromosome segments were found. *Note* There were differences in chromosomal banding levels because the patient and his mother did not undergo cytogenetic testing in the same laboratory

G-banding revealed a derivative X chromosome: add(X)(p22) (Fig. 2a). After C-banding verification, it was found that a long heterochromatin region existed in the unknown segment of der(X) (Fig. 2c). No abnormal findings in FISH analysis. CMA showed arr[GRCh38] Xp22.33(251888_1772154) × 1 (1.52 Mb) (Fig. 2e), and no origin of heterochromatin was detected, nor *SRY* or *AZF* genes were identified. Pedigree genetic analysis (Fig. 1) for members with the same clinical manifestations revealed that her mother’s (Fig. 1, II-8) G-banding karyotype (Fig. 2b) and her cousin’s (Fig. 1, III-2) CMA results (Fig. 2f) were the same as hers (Fig. 1, III-4). The patient’s father (Fig. 1, II-7) and sister (Fig. 1, III-5), who had no clinical manifestations, showed normal karyotypes. Her maternal grandfather and grandmother had died and could not be traced. Combined with genetic and clinical analyses, the karyotype and array results were thus interpreted as: 46,X,der(X)t(X;Y)(p22.33;q12)mat.arr[GRCh38] Xp22.33(251888_1772154) × 1 (1.52 Mb), whereas the X deleted segment harbored the short-stature homeobox (*SHOX*) gene. Since the Affymetrix CytoScan 750 k chip used in this laboratory had no probe coverage in the Yq12 region, it was impossible to detect the translocation of this Y chromosome segment; however, combined with the large heterochromatin region shown in the chromosome C-banding results, we suspect that this heterochromatin segment was from the Y chromosome Yq12 region.

**Fig. 3:**
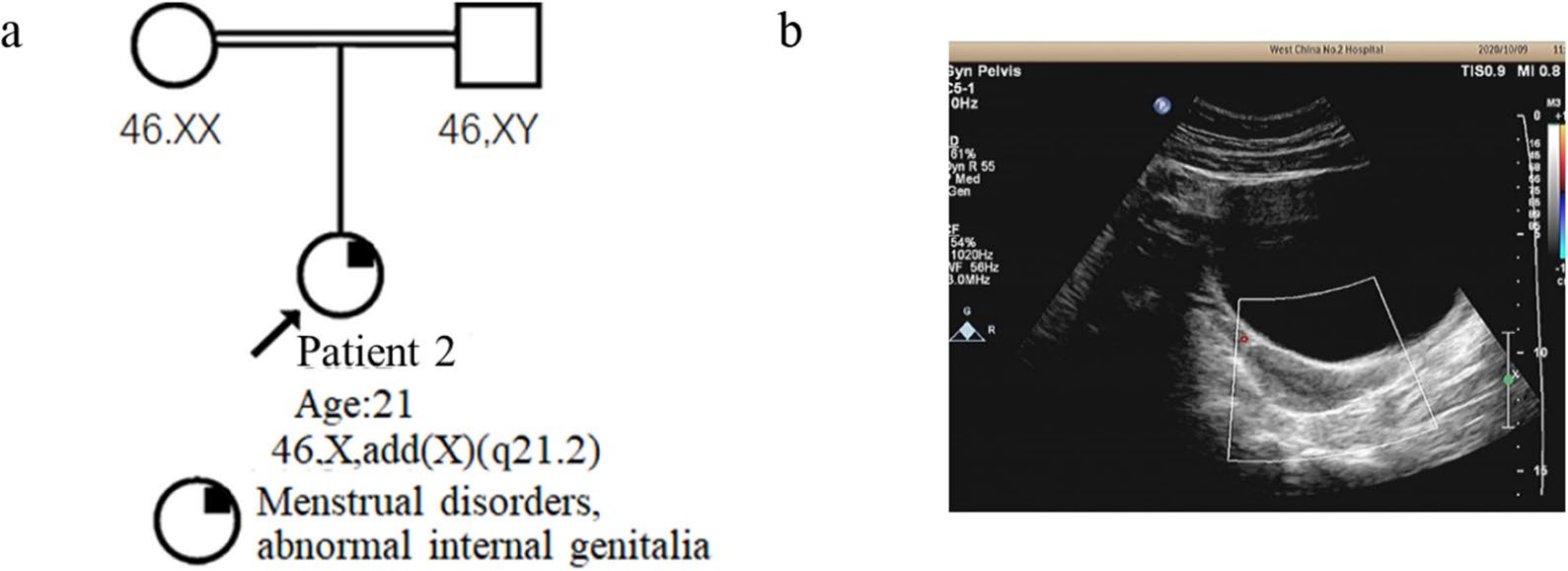
Pedigree and gynecological ultrasound results of patient 2. **a**: The pedigree shows that the patient’s parents are consanguineous and the G-banding karyotype of both parents is normal; **b**: Uterine and appendage ultrasound shows a small uterus, and the bilateral ovaries are unclear

*Patient 2* was a female (age: 21 years, height: 161 cm, and weight: 57 kg) who had visited our hospital for menstrual disorders for five years and secondary amenorrhea. Menarche began at 13 years of age, menstruation was normal from 13 to 15 years of age, and menstrual thinning started at 15 years of age (1–2 months). Thinning gradually spaced out and amenorrhea developed at 17 years of age. She reported that at the age of 16 years, she was suspected of having polycystic ovary syndrome (PCOS) and was treated symptomatically. At 18 years of age, ultrasonography suggested gonadal dysgenesis (GD). The patient was the only child in her family. Her parents were consanguineous (Fig. 3a), in a good health, and had no similar family history. Gynecological examination revealed internal genital abnormalities. Ultrasonography at 21 years of age showed that the uterus was small and both ovaries were streaks (Fig. 3b). Sex hormone testing showed E2: < 11.8 (low level); P: 0.23; LH: 24.2; FSH: 68.9 (high level); and AMH (ELISA): < 0.06 ng/ml. The patient is currently being treated with hormone replacement therapy (HRT). Clinical materials and laboratory test results are summarized in Table 1.

**Fig. 4:**
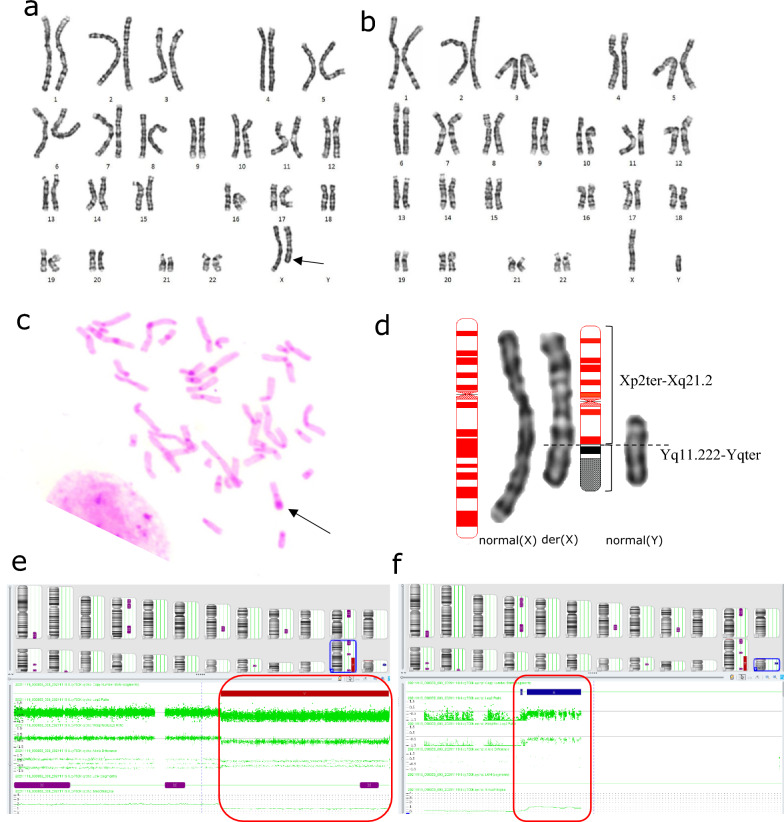
Cytogenetic and CMA results of patient 2. **a**: The results of the G-banded karyogram (550 bands) of patient 2(a derivative X chromosome is indicated by an arrow); **b**: The result of the G-banded karyogram (550 bands) of the patient’s father: a normal male karyotype; **c**: Fragment of C-banded metaphase spread of patient 2 (photomicrography), showing a large heterochromatin region at the end of the long arm of derivative X chromosome; **d**: patient 2’ X chromosome (left) and her father’s Y chromosome (right) comparison chart, the results show that the Xq21.2 (of der(X)) unidentified source segment of the band are more consistent with the father’s Yq11.2 band; **e, f**: CMA results show that a large 69.689 Mb deletion of the long arm of the X chromosome(red box in e), and a partial duplication of the long arm of the Y chromosome (red box in f), as well as multiple regions with AOH (red regions in the figure)

The G-banding karyotype showed a derivative X chromosome: add(X)(q21) (Fig. 4a). After verification by C-banding, a long heterochromatin region was found in the unknown segment of der(X) (Fig. 4c). No abnormal findings in FISH analysis. The CMA results were as follows: arr[GRCh38] Xq21.2q28(86025630_155714301) × 1 (69.689 Mb) Yq11.222q12(18443276_26653507) × 1 (8.21 Mb). There were large genomic homozygous regions (Fig. 4e, f) and the *SRY* and *AZF* genes were not identified. Karyotype analysis of her parents showed no abnormalities (father’s karyotype Fig. 4b). The karyotype and array results were thus interpreted as: 46,X,der(X)t(X;Y)(q21.2;q11.2)dn.arr[GRCh38] Xq21.2q28(86025630_155714301) × 1, Yq11.222q12(18443276_26653507) × 1.

**Fig. 5:**
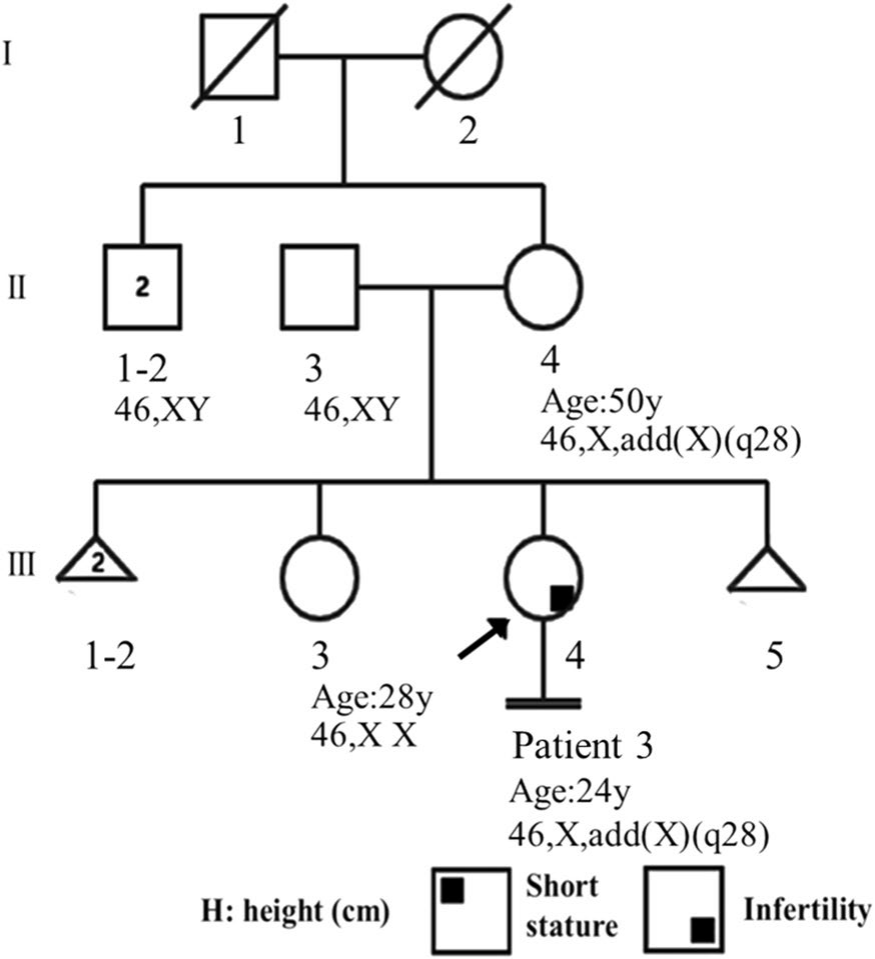
Pedigree of patient 3. Six family members underwent G-banding karyotyping; the mother’s karyotype results were consistent with patient 3, and the other results were normal. The sex of the three aborted embryos is unknown

*Patient 3* (Fig. 5, III-4) was a female (age: 24 years, height: 150 cm, and weight: 54 kg) who visited our hospital for infertility. She had a normal sexual life and a history of surgery for an ovarian cyst at 18 years of age and bilateral tubal ligation due to bilateral hydrosalpinx at 23 years of age. No special facial features or menstrual histories were noted. Her parents were not consanguineous and the mother (Fig. 5, II-4) had had three miscarriages (Fig. 5, III-1, III-2, III-5). The older sister (Fig. 5, III-3) was 28 years old, and her menstruation was normal. Recent ultrasonography showed that the uterus and ovaries were normal, and sex hormone testing revealed E2: 102.3; P: 15.29; LH: 5.1; FSH: 3.4; and AMH (ELISA): 3.97 ng/ml. The clinical data and laboratory test results are summarized in Table 1.

**Fig. 6:**
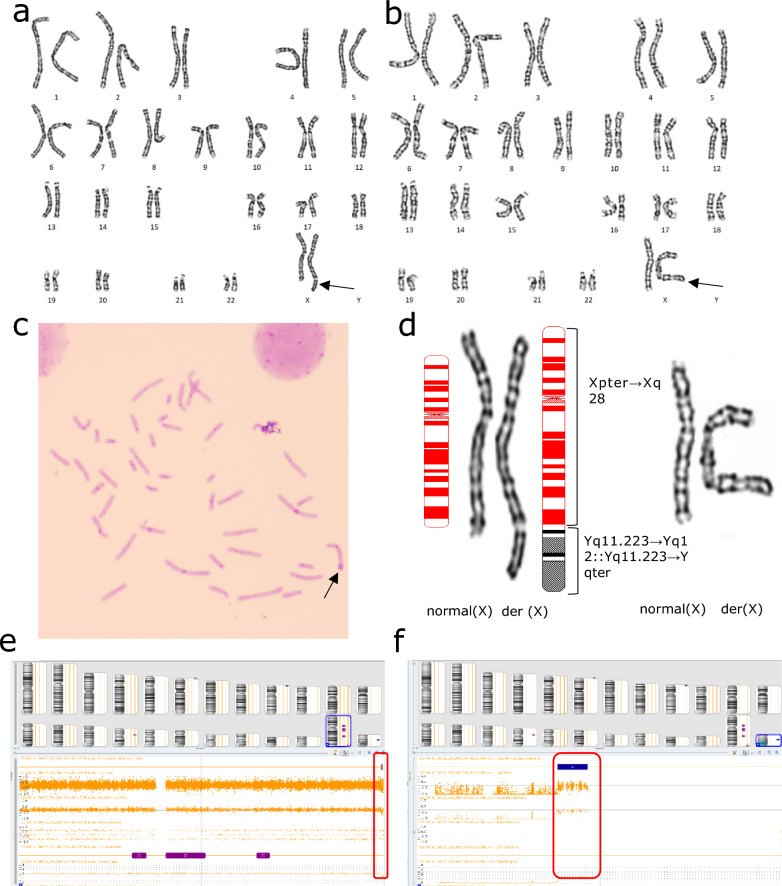
Cytogenetic and CMA results of patient 3 and abnormal X chromosome. **a:** The results of the G-banding (550 bands) of patient 3, there is a derivative X chromosome (arrow); **b:** The mother’s G-band karyotype (550 bands) shows the same der(X) (arrow) as in patient 3; **c:** C-banding of patient 3, showing larger heterochromatin segments in the long arm end segments of the der(X); **d:** Comparison of X chromosomes of patient 3 (left) and her mother (right). **e, f:** CMA results showing deletions of large segment at the end of the long arm of chromosome X (red box in e) and two copies of the segments of the long arm part of the chromosome Y (red box in f)

G-banding karyotyping showed a derivative X chromosome: add(X)(q28) (Fig. 6a). A long heterochromatin region existed in the unknown region of the derived X chromosome (Fig. 6c). No abnormal findings in FISH analysis. The CMA results were as follows: arr[GRCh38] Xq28(155077922_155700385) × 1 (0.622 Mb),Yq11.223q12(21924024_ 26,653,507) × 2 (4.729 Mb) (Fig. 6e, f). and no *SRY* or *AZF* genes were detected. The cytogenetic analysis of the patient’s mother (Fig. 5, II-4) was consistent with that of the proband (Fig. 6b). For the patient’s father (Fig. 5, II-3), sister (Fig. 5, III-3), and mother’s siblings (Fig. 5, II-1, II-2), the chromosomal karyotyping analysis did not show obvious abnormalities. The karyotype and array results were thus interpreted as: 46,X,der(X)t(X;Y)(q28;q11.223)t(Y;Y)(q12;q11.223)mat.arr[GRCh38] Xq28(155077922_155700385) × 1, Yq11.223q12(21924024_26653507) × 2.

### Literature review and reclassification

A total of 81 articles were retrieved, where 128 patients with X/Y translocation were reported, of which, approximately 32 articles analyzed the core family. According to Hsu’s report in 1994 [1], X/Y translocations are divided into 11 types (7+4), and the most common types are types 1 and 2. In our study, patients were reclassified according to the location of the breakpoints in the translocated X and Y chromosomes and the centromeric composition of the derived chromosomes. Patients with breakpoints located at Xp22 were categorized into five types (types I-V) based on the composition of the sex chromosome centromere. Type I/type II/type III are consistent with Hsu’s type 1/type 2/type 3. Patients with 46,X,der(X)t(X;Y)(p22;p11) were categorized into type IV, and Hsu’s type A/type B were merged into type V. Patients with breakpoints located outside of Xp22 were classified as type “other”. The reclassifications and phenotypes are summarized in Tables 2, 3, 4.

**Table 2:** Summary of the two classifications

New types			Hsu’s types	
Basis	Type	Karyotype	Type	Karyotype
Xp22	type I	46,Y,der(X)t(X;Y)(Xqter → Xp22::Yq11 → Yqter)	type 1	46,Y,der(X)t(X;Y)(Xqter → Xp22::Yq11 → Yqter)
	type II	46,X,der(X)t(X;Y)(Xqter → Xp22::Yq11 → Yqter)	type 2	46,X,der(X)t(X;Y)(Xqter → Xp22::Yq11 → Yqter)
	type III	46,X,psu dic(X;Y)(Xqter → Xp22::Yp11 → Yqter)	type 3	46,X,psu dic(X;Y)(Xqter → Xp22::Yp11 → Yqter)
	type IV	46,X,der(X)t(X;Y)(Xqter → Xp22::Yp11 → Ypter)	type 4	46,X,der(X)t(X;Y)(Xqter → Xp11.2::Yq11 → Yqter)
	type V	46,X,der(Y)t(X;Y)(Ypter → Yq11::Xp22 → Xpter)	type 5	46,X,der(X)t(X;Y)(Xpter → Xq22::Yq12 → Yqter)
Not-Xp22	type other	–	type 6	46,X,der(X)t(X;Y)(Xpter → Xq22::Yq11 → Yqter)
			type 7	46,X,psu dic(X;Y)(Xpter → Xq22::Yp11 → Yqter)
			type A	46,X,der(Y)t(X;Y)(Ypter → Yq11.2::Xp22.1 → Xpter)
			type B	46,X,der(Y)t(X;Y)(Ypter → Yq11.23::Xp22.1 → Xpter)
			type C	46,X,der(Y)t(X;Y)(Ypter → Yq11.21::Xp21 → Xpter)
			type D	46,X,der(Y)t(X;Y)(Ypter → Yq11.23::Xq28 → Xqter)

**Table 3:** Summary of reported Xp22; Yq11 and Xp22; Yp11 translocation

The exception type		Type I		Type II		Type III		Type IV		Type V	
	Gender	M	F	M	F	M	F	M	F	M	F
Category	Number	33	0	2	53	2	2	7	2	6	0
Origin	Pat	0	0	0	1	0	0	0	0	0	0
	Mat	29	0	0	8	0	0	0	0	0	0
	De novo	0	0	1	8	1	0	2	1	2	0
	Unknown	4	0	1	36	1	2	5	1	4	0
Stature	Short	26	0	0	42	2	1	4	0	2	0
	Normal	6	0	2	10	0	0	3	1	0	0
	Unknown	1	0	0	1	0	1	0	1	4	0
Limbs	Short	12	0	0	8	0	0	2	1	4	0
	Normal	21	0	2	45	2	2	5	1	2	0
	Unknown	0	0	0	0	0	0	0	0	0	0
Gonad	Abnormal	10	0	1	9	2	1	3	0	3	0
	Normal	1	0	0	27	0	0	1	1	0	0
	Unknown	22	0	1	17	0	1	3	1	3	0
Psychomotor movements	Abnormal	10	0	0	4	0	0	0	0	3	0
	Normal	22	0	2	49	2	2	7	1	3	0
	Unknown	1	0	0	0	0	0	0	1	0	0
Mental	Abnormal	13	0	0	4	0	0	0	0	2	0
	Normal	17	0	2	47	2	2	7	1	4	0
	Unknown	3	0	0	2	0	0	0	1	0	0
Facial deformities	P	15	0	1	7	0	0	1	0	3	0
Eye abnormalities	P	1	0	0	2	0	0	0	1	0	0
Ichthyosis	P	12	0	0	1	0	0	1	0	0	0
Chondrodysplasia punctata	P	6	0	0	1	0	0	1	0	0	0
Leri-Weill dyschondrosteosis	P	9	0	0	4	0	1	1	0	2	0

*M* male, *F* female, *P* positive, patients present with this clinical phenotype, *Pat* paternal, *Mat* maternal. Unknown: There is no mention of the relevant circumstances in the text

**Table 4:** Summary of type “other” (new) for Y/X translocation

Detailed results	Gender	Short stature	Mental retard-ation	Head and face retard-ation	Limbs retard-ation	Optic abnormalities	L-W Chondrosclerosis	Psychomotor abnormalities	Gonad abnormalities
46,X,der(X)t(X;Y)(q22;q11)dn [5]	F	−	−	−	−	−	−	−	+
46,X,psu dic(X)t(X;Y)(q22;p11) [6]	F	−	−	−	−	−	−	−	+
46,X,der (X)t(X;Y)(p11.2;q11)dn [27]	F	+	−	−	−	−	−	−	+
46,X,der(X)t(X;Y)(q25;q12) [28]	F	−	−	−	−	−	−	−	+
46,X,der(X)t(X;Y)(q26.2;q11.223) [29]	F	−	−	−	−	−	−	−	+
46,X,der(X)t(X;Y)(q26.2;q11.223)mat [29]	F	−	−	−	−	−	−	−	+
46,X,der(X)t(X;Y)(q26.3;q11.223) [30]	F	n	−	−	−	−	−	−	−
46,X,der (X)t(X;Y)(q28;q11.2)dn [31]	F	−	−	−	−	−	−	−	+
46,X,der(Y)t(X;Y)(p21.1;q11)dn [32]	F	+	+	+	−	−	−	−	n
46,X,der(Y)t(X;Y)(p21.2;p11.3) [33]	F	+	−	+	−	−	−	+	+
46,X,der(Y)t(X;Y)(q13.1;q11.223) [34]	F	−	−	−	−	−	−	−	+
47,XY,der(Y)t(X;Y)(p21.1;p11.2)* [35]	F	n	−	+	−	+	−	−	+
46,X,der(Y)t(X;Y)(q28;q11.23)dn [36]	M	−	−	+	−	−	−	+	+
46,X,der(Y)t(X;Y)(p21.3;q11.21) [36]	M	−	−	+	+	+	+	+	+
46,X,psu dic(X;Y)(p11.3;p11.1) [37]	F	+	−	−	−	−	−	−	n
47,X,der(X)t(X,Y)(p11.4,p11.2)X2[65%]/46,X,der(X)(p11.4,p11.2)[35%]mat [38]	F	+	−	−	−	−	−	−	n
45,X[25%]/46,X,der(X)t(X;Y)(p11.4,p11.2)[75%] [37]	F	+	−	−	−	−	−	−	−
46,X,psu dic(X;Y)(p22;q11)dn [38]	M	+	−	−	−	−	−	+	n
45,der(X)t(X;Y)(p22.3;p11.2)[8]/46,t(X;Y)(p22.3;p11.2)[12] [7]	M	−	−	−	−	−	−	−	+
46,X,dic(X;Y)(p22.33;p11.32)/45,X/45,dic(X;Y)(p22.33;p11.32) [8]	M	−	n	−	−	−	−	−	+
45,der(X)t(X;Y)(p22;p11.3)ins(X;Y)(p22;q12)[[80]/45,X[20] [40]	M	+	−	+	−	−	−	−	n

“+”: with an exception in this regard; “−”: there is no anomaly in this regard; n: this aspect is not described

Type “other” include types 4\5\6\7, type C\D and other karyotypes

*M* male, *F* female

^*^: This patient is a sex reversal case report

The patients belonging to type I [46,Y,der(X)t(X;Y)(p22;q11)], having complete absence of X(pter → p22) and two copies of Y(q11 → qter), one on the X chromosome and one as a normal Y, were all males (33/33). In these patients, the der(X) was inherited from their mother (30/33) or was of unknown origin (3/33). Most type I patients had abnormal clinical manifestations, such as short stature and limb anomalies [22], intellectual disability [11], and may have gonadal abnormalities (eg. cryptorchidism) [22, 23]***.*** Those with type II [46,X,der(X)t(X;Y)(p22;q11)], type III [46,X,psu dic(X;Y)(p22;p11)], and type IV [46,X,der(X)t(X;Y)(p22;p11)] had an X(pter → p22) single copy. In type II patients the majority were females and have a single copy of Y(q11 → qter)(53/55).Types III and IV patients had a single copy of Y(p11 → qter) or Y(p11 → pter), respectively. The phenotypic sex of reported patients depends on the presence of SRY gene, however, three patients (one from type III and two from type IV) were presenting with a female phenotype despite having SRY gene [24–26]. Type V [46,X,der(Y)t(X;Y)(p22;q11)] patients had X(p22 → qter) and Y(pter → q11) single copies, and an extra copy of X(p22 → pter), and all patients were males (6/6). The details are presented in Table 3.

Type “other” included 21 patients, of which, 15 were females and 6 were males. Patients with type “other” were more likely to have gonadal abnormalities (14/21). Almost all patients had at least one complete X-chromosome segment (details shown in Table 4) [5–8, 27–40].

## Discussion

The inheritance of structurally altered X and Y chromosomes differs from that of autosomes, and it is difficult for X/Y balanced translocation carriers to have a descendant with a balanced chromosomal structure. Translocation between the X and Y chromosomes can impact the pairing of sex chromosomes during meiosis and result in different patterns of X chromosome inactivation [41, 42], which can affect growth and development.

In the Patient 1 with X chromosome deletion followed by der(X)t(X;Y), the deleted region (Xp22.3) contains *SHOX* gene required for normal bone formation. Its haplo-insufficiency can result in short stature and Leri-Weill dyschondrosteosis, which usually has a more severe phenotype in females [43, 44]. The patient (Fig. 1, III-4), her mother (Fig. 1, II-8), and cousin (Fig. 1, III-2) showed symptoms of short stature. As no genetic testing was available for male patients in this family, it was impossible to identify whether the short male patients in the family (Fig. 1, II-2, II -9) and the cousin (Fig. 1, III-6) carried the same der(X); however, based on the pedigree, we speculated that the original patient (Fig. 1, I-2) may have passed the der(X) to her sons (Fig. 1, II-2, II-9) and daughters (Fig. 1, II-5, II-8), then to her granddaughters (Fig. 1, III-2, III-4, III-6). The proband’s female-specific hormones had no significant abnormalities, except for a decrease in AMH to 0.98 ng/ml, indicating a mild decrease in ovarian reserve function. After a year of treatment and good condition in daily life, the latest AMH level was 1.96 ng/ml, indicating that her ovarian reserve function showed a good recovery. Her mother had given birth to two children, with no history of miscarriage, and began experiencing irregular menstruation and prolonged menstrual cycles around the age of 40 years. She did not undergo hormonal profiling, so the possibility of premature ovarian failure (POF) cannot be ruled out. Other women with short stature in this family (Fig. 1, II-5, II-8), who may carry der(X), have all been pregnant. Patient 1 and her cousin still have a chance of becoming pregnant, although they experienced recurrent spontaneous abortion (RSA) (Fig. 1, III-2) or infertility (Fig. 1, III-4), which may be result from recombination changes of der(X) during meiosis. Genetic counseling was provided for the patient with information on the nature, mode of inheritance, the genetic risk for offspring, and prenatal testing issues. Currently, Patient 1 has pregnancy desire and assisted reproductive technology (ART) has been proposed for the infertility treatment.

**Fig. 7:**
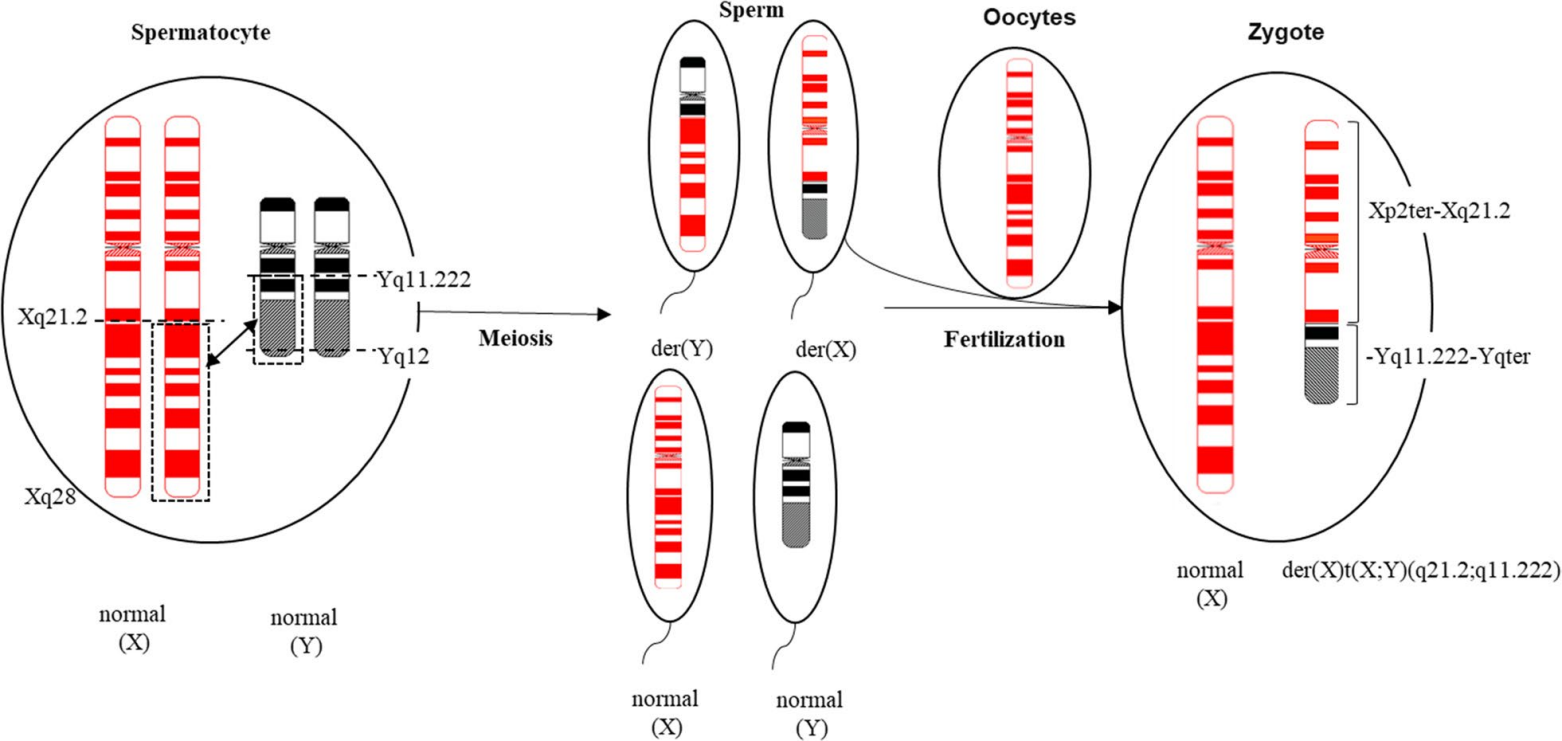
The chromosomes X and Y pair and recombine at the meiosis, and fertilization. It is speculated that the patient 2’s der(X) is derived from the abnormal recombination of X chromosome Xq21.2 and Y chromosome Yq11.222 during meiosis of paternal spermatocytes, with an idiogram of 550 band

Patient 2 exhibited X chromosome deletion (q21.2-qter)(86025630_155714301) × 1 and translocated Y(q11.2-qter)(18443276_26653507) × 1 region followed by der(X)t(X;Y). The deleted region X(q21.2-q28) included multiple dosage-sensitive genes that may cause diseases. The Y region contains AZF factors that are required for spermatogenesis that are not relevant to the female phenotype. The parents were consanguineous, and their karyotypes were normal; therefore, the source of der(X) was an abnormal recombination between X and Y during the first paternal meiosis [7], forming a sperm with a derivative X (Fig. 7). The patient had abnormal reproductive system development, including a small uterus and streak ovaries, and her sex hormones showed relatively high levels of FSH and low E2. The AMH level (ELISA) was extremely low, indicating ovarian dysfunction. Currently, across eight articles, nine patients had the Xq/Yq translocation, including a family study where phenotypes varied in severity and included menstrual disorders, primary/secondary amenorrhea, POF, streak gonads, and non-pregnant chorionic carcinoma [29, 31]. Studies by Tharapel et al. showed that deletion of different regions of the long arm of the X chromosome can be associated with GD or POF. Women with X(q13-qter) region deletion were more likely to develop complete ovarian failure, whereas women with X(q24-qter) deficiency may have symptoms of POF [45]. Researchers have speculated that X(q13-q26) is the “critical area” for ensuring normal ovarian function, which conforms to the observations made for Patient 2 [46, 47]. Baronchelli et al. studied a family in which the daughter’s der(X)t(X;Y)(q26.2;q11.223) came from the mother, and both the mother and daughter had symptoms of POF [29]. This indicated that it is still possible to have children with X(q26.2-qter) deletion. However, the breakpoint in our patient is more proximal and thus it was associated with a more severe phenotype. Premature ovarian failure 2A (POF2A) was described to result from deletion or mutation in the *DIAPH2* gene on chromosome Xq22 (OMIM# 300108). The patient’s parents were consanguineous, and CMA indicated multiple regions with absence of heterozygosity (AOH), which did not completely exclude the patient’s clinical correlation with these regions. Genetic counseling was provided for the patient with information on the nature, and the risk of implications about gonadoblastoma. Patient 2 was now treated with HRT and followed up clinically.

Patient 3 had X chromosome deletion (Xq28)(155077922_155700385) × 1 followed by der(X)t(X/Y). Xq28 is a gene rich region that contains many pathogenic loci that have X-linked recessive inheritance. The patient’s mother (Fig. 5, II-14) also carried the same der(X), with regular menstruation and menopause at 50 years of age and without abnormal phenotype. The 0.622 Mb deletion region in our patient did not involve POF susceptibility genes. Therefore, Patient 3 and her mother did not have manifestations of ovarian dysfunction. Family analysis revealed that her mother (Fig. 5, II-4) had three spontaneous abortions (Fig. 5, III-1, III-2, III-5), and did not give birth to a boy. The mother’s X chromosome origin could not be traced, as her maternal grandfather (Fig. 5, I-1) and grandmother (Fig. 5, I-2) had passed away, and both uncles (Fig. 5, II-1, II-2) had a normal male karyotype. However, Delon et al. reported a karyotype of 46,X,der(X)t(X;Y)(q28;q11.2)dn in a 28-year-old female patient with infertility similar to our Patient 3 [31]. Therefore, based on the pregnancy history of the patient’s mother, we speculated that this der(X) may undergo recombination changes during meiosis, which results in the failure to produce normal embryos. Alternatively, multiple pathogeneic genes in the deletion region of the X chromosome, such as *RAB39B* (OMIM 300774) and *TMLHE* (OMIM 300777) have an XLR inheritance and nullosomy of which can lead to a severe phenotype in the male fetus, causing miscarriage or embryo lethality [48]. The gained Y(q11.223q12)segment in which no *SRY* or *AZF* genes were detected; therefore, this region has no clinical correlation in this female patient. Genetic counseling was provided for the patient with information on the nature, the genetic risk for offspring and prenatal testing issues. Currently, the patient has pregnancy desire and ART has been proposed because her bilateral fallopian tubes have undergone ligation surgery.

In most of studies related to X/Y translocation (Tables 3 and 4), breakpoints in the X/Y translocation are located in Xp22/Yq11 (type I/type II/type V, approximately 77.19%). However, it is worth mentioning that only one patient with definitive childbearing has been reported among all male patients [9], while many women have had children. Patients with Xp22/Yq11 translocations tend to complain of short stature. Some patients with the Xp22/Yq11 translocation also have Leri-Weill dyschondrosteosis, which is characterized by Madelung deformity. Male patients may also have contiguous gene deletion syndrome manifestations, such as intellectual disability, skeletal anomalies, psychomotor abnormalities, gonadal abnormalities, facial deformities, chondrodysplasia punctata (ARSE gene deletion) [49], ichthyosis (STS gene deletion) [50], and other symptoms, which are usually absent or less intense in female patients [3].

A small number of breakpoints were located at Xp22/Yp11 (type III/type IV, approximately 8.77%). Although a bias may occur due to the small number of patients, it is interesting to note that none of the patients with Xp22/Yp11 translocation had significant signs of deformity, except for one patient with induced fetal abortion due to an upper extremity abnormality [23]. Male patients with Xp22/Yp11 translocation in types III and IV have karyotypes of 46,X,psu dic(X;Y)(p22;p11) and 46,X,der(X)t(X;Y)(p22;p11) and 45,der(X)t(X;Y), respectively. Both types have a deletion of the chromosome Y segment, resulting in phenotypic effects related to disorders of sexual development. Three female patients with type III and type IV had verified *SRY* gene in der(X) [24–26]. This could be partly explained by the inactivation of der(X), which may spread into the translocated Yp segment, resulting in the *SRY* gene expression being suppressed or completely inhibited [33, 35, 42, 51]. Female patients with type IV had milder phenotypes than those with other types, indicating that Yp may compensate for the Xp22 partial gene deletions, which also matches the presence of a pseudoautosomal region (PAR1) in Xp and Yp [52, 53]. However, they still had gonadal abnormalities, which is consistent with previous studies showing that two normal X chromosomes are essential for normal ovarian function [54].

Breakpoints rarely occur at other locations such as Xp21, Xp11, and Xq (Table 4, approximately 14%), and the karyotype is mostly 46,X,der(X)/46,X,der(Y) among female patients. The two reported male patients had severe phenotypic effects [36], illustrating that the insufficient gene dosage of chromosome X may affect male survival. The incidence of gonadal abnormalities was significantly higher in female patients.

All patients in the above classifications contained at least one normal X chromosome, except for those with type I. Patients with type I also had a higher probability of nervous, gonadal, and bone dysplasia than those with other types, indicating that the complete human genome is essential for human development.

## Conclusion

X/Y translocation is complex in both clinical presentation and classification, and we reviewed this category of cases by performing systematic clinical and cytogenetic analyses of three patients with different phenotypic presentations. This information will further improve understanding of the clinical and genetic aspects of X/Y translocations.

## Data Availability

All data generated or analyzed during this study are included in this published article and its supplementary information files.

## Abbreviations

FISH: Fluorescence in situ hybridization
CMA: Chromosomal microarray analysis
PGT: Preimplantation genetic testing
E2: Estradiol
P: Progestone
LH: Luteinizing hormone
FSH: Follicle-stimulating hormone
AMH: Anti-Müllerian hormone
ELISA: Enzyme linked immunosorbent assay
GD: Gonadal dysplasia
PCOS: Polycystic ovary syndrome
ART: Assisted reproductive technology
